# Obituary

**Published:** 1851-05

**Authors:** 


### OBITUARY.

Professor John B. Beck departed this life, in the city of New York, on the 9th day of April. He was 57 years of age, and had long enjoyed an enviable position in the medical profession. We learn from the New York Journal of Medicine, that Professor Beck occupied a professorial chair in the College of Physicians and Surgeons, for almost twenty-five years. Of the distinguished ability with which he filled it, the medical profession of the Union are well advised. The profession of the city of New York, especially will feel the loss of Professor Beck, but his death is a calamity to the country.

The *New York Journal of Medicine* promises a biographical memoir of the deceased, in its July number, and we shall endeavor to present our readers, at least a synopsis of it. B.

